# Double Pathology: Malignant Epithelial Ovarian Tumor and Germ Cell Tumor (Choriocarcinoma), a Rare Coexistence

**DOI:** 10.14740/wjon848w

**Published:** 2015-08-27

**Authors:** Gouthaman Shanmugasundaram, Elilnambi Sundaramoorthy, Suresh Sudalaiandi, Satish Srinivas Kondaveeti, Thanka Johnson, Rajendiran Swaminathan, Anita Ramesh

**Affiliations:** a Department of Surgical Oncology, Sri Ramachandra Medical College and Research Institute, Sri Ramachandra University, Porur, Chennai, India; b Department of Medical Oncology, Sri Ramachandra Medical College and Research Institute, Sri Ramachandra University, Porur, Chennai, India; c Department of Oncology, Sri Ramachandra Medical College and Research Institute, Sri Ramachandra University, Porur, Chennai, India; d Department of Pathology, Sri Ramachandra Medical College and Research Institute, Sri Ramachandra University, Porur, Chennai, India

**Keywords:** Epithelial ovarian carcinoma, Choriocarcinoma, Germ cell tumor

## Abstract

Surface epithelial stromal tumors account for approximately 60% of all ovarian tumors and approximately 90% of all ovarian malignancies. Sex cord stromal tumors account for 7% of all malignant ovarian tumors. Germ cell tumors make up only 3-7% of malignant ovarian tumors. A combination of serous carcinoma of the ovary and choriocarcinoma is rare. Until today such combination has been documented only in six cases in the English literature. Here, we describe a case of ovarian serous carcinoma, where histopathology revealed a combination of serous carcinoma with adjacent choriocarcinoma component in the extraovarian peritoneal deposits. A 64-year-old post-menopausal female was diagnosed to have stage IV ovarian cancer. She received six cycles chemotherapy. Subsequently she underwent optimal cytoreductive surgery. Microscopically, monomorphic histology (serous carcinoma) was noted in both the ovaries and dimorphic histologies (serous carcinoma and choriocarcinoma) in the sigmoid mesocolon nodule, omentum and left subdiaphragmatic nodules. Metronomic chemotherapy continued and patient is on regular follow-up for the past 1 year with stable disease. Recognition of choriocarcinomatous components in ovarian carcinomas is important because of its association with aggressive behavior. In spite of the aggressive histology, the patient is surviving for the past 1 year. Different chemotherapeutic regimens have been used in cases of mixed choriocarcinoma and carcinoma, but established chemotherapeutic regimens have not been described. Chemotherapeutic regimens that target both components have been advocated and used. The absence of choriocarcinoma in ovarian primary and its presence in the extraovarian peritoneal deposits have not been described in the English literature so far. This case is being presented for its rarity.

## Introduction

Epithelial ovarian carcinoma comprises more than 90% of all ovarian malignancies, and because symptoms are commonly only vague and non-specific, most cases present at an advanced stage with extensive extraovarian involvement [1]. Most tumors of the ovary can be placed into one of three major categories: surface epithelial stromal tumors, sex cord stromal tumors and germ cell tumors. Surface epithelial stromal tumors account for approximately 60% of all ovarian tumors and approximately 90% of all ovarian malignancies. Sex cord stromal tumors account for 7% of all malignant ovarian tumors. Germ cell tumors make up only 3-7% of malignant ovarian tumors [2]. A combination of serous carcinoma and choriocarcinoma is rare. Until today such combination has been documented only in six cases in the English literature [3-7]. Hence, we describe a case of ovarian serous carcinoma, where histopathology revealed a combination of serous carcinoma with adjacent choriocarcinoma component in the extraovarian peritoneal deposits. The absence of choriocarcinoma in ovarian primary and its presence in the extraovarian peritoneal deposits have not been described in the English literature so far.

## Case Report

**Figure 1:**
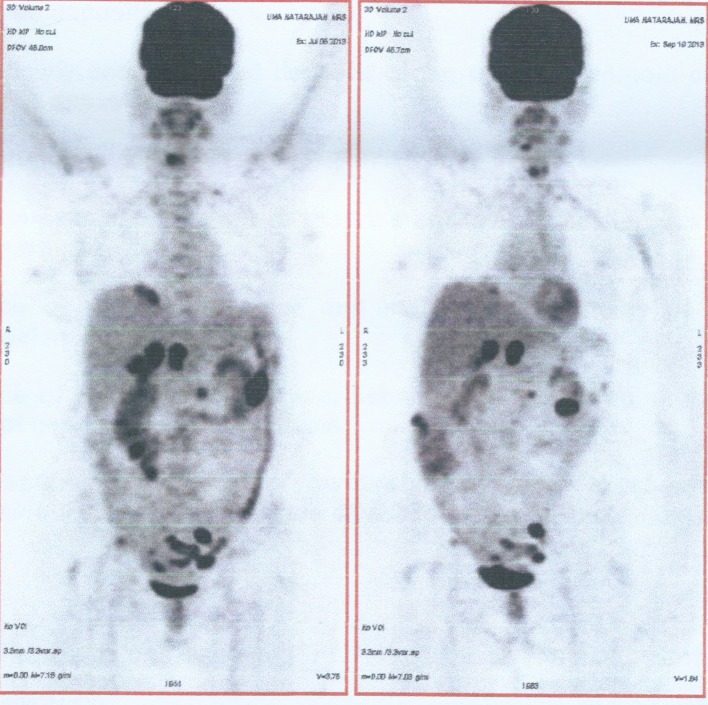
Comparision of whole body FDG PET CT scan before and after chemotherapy.

A 64-year-old post-menopausal female presented to our hospital with belching and bloating sensation. The patient had undergone vaginal hysterectomy 8 years back for a benign condition. Physical examination revealed gross ascites. On investigation, CA125 was 1,240 U/mL. Whole body PET CT scan revealed metabolically active lesions in both adnexa, multiple peritoneal lesions, retroperitoneal nodes, mediastinal nodes and left supraclavicular nodes. Ascitic fluid cytology was positive for malignant cells. She was diagnosed to have stage IV ovarian cancer. She received six cycles chemotherapy: initial three cycles of cisplatin and paclitaxel and subsequently three cycles of carboplatin and paclitaxel, as there was toxicity to cisplatin. Whole body PET CT scan at the end of six cycles of chemotherapy showed regression in metabolic activity of adnexal lesions, retroperitoneal nodes and supraclavicular nodes with no metabolic activity in mediastinal nodes and reduction in CA125 levels to 16 U/mL (Fig. 1). Subsequently she underwent optimal cytoreductive surgery including bilateral ovariectomy, total omentectomy, bilateral pelvic lymph node dissection and excision of all macroscopic peritoneal nodules except for < 1 cm multiple nodules in the bilateral subdiaphragmatic peritoneum. Postoperative period was uneventful.

**Figure 2:**
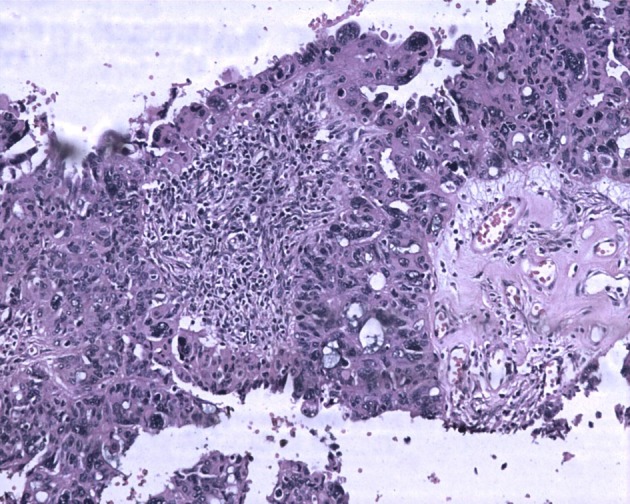
Ovarian tumor with focal papillary pattern. The tumor cells are large, pleomorphic with abundant eosinophilic cytoplasm. Hematoxylin and eosin, × 200.

**Figure 3:**
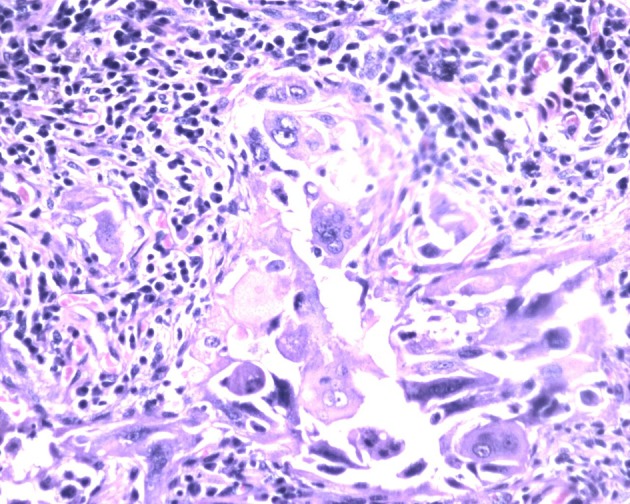
Biphasic tumor cells with multinucleated syncytiotrophoblasts and mononucleated cytotrophoblast surrounded by lymphocytes and plasma cells. Hematoxylin and eosin, × 400.

Macroscopically right ovary measured 4 × 2 × 1 cm and the left was 4 × 3 × 1 cm. Cut sections of the ovaries revealed solid areas with focal necrosis (20%). Largest peritoneal nodule over sigmoid mesocolon measured 1.5 × 1 × 0.8 cm. Largest left subdiaphragmatic nodule was 1 × 0.8 × 0.4 cm. Microscopically, monomorphic histology was noted in both the ovaries and dimorphic histologies in the sigmoid mesocolon nodule, omentum and left subdiaphragmatic nodules. One histology showed solid sheets and cohesive highly pleomorphic neoplastic cells with occasional formation of acini, slit like spaces and papillae suggestive of high grade papillary serous carcinoma (Fig. 2). The second histology demonstrated solid aggregates of neoplastic cells with a biphasic morphology consisting of cytotrophoblasts admixed with multinucleated syncytiotrophoblasts, a picture characteristic of choriocarcinoma (Fig. 3). Because of this rare combination, immunohistochemistry for beta-HCG was performed in the areas showing choriocarcinomatous area, but it was negative. Serum beta-HCG estimated postoperatively was < 2.0 IU/mL. Patient was advised for further chemotherapy in view of residual disease; however, she was unwilling for intravenous chemotherapy and hence started on oral chemotherapy (endoxan and topotecan), drugs which are also active against choriocarcinoma. Metronomic chemotherapy continued and patient is on regular follow-up for the past 1 year with stable disease.

## Discussion

The standard treatment for women with advanced stage ovarian tumor, if operable, has been hysterectomy and bilateral salpingo-oophorectomy and staging biopsies, including omentectomy, peritoneal biopsies, pelvic and para-aortic lymph node dissection, followed by platinum-based combination chemotherapy [8, 9]. Some patients with a poor performance status due to extensive tumor or comorbid disease may not be appropriate candidates for this procedure. In these cases, initiating neoadjuvant chemotherapy, followed by an interval attempt at surgical cytoreduction, is a reasonable approach [10, 11].

Malignant surface epithelial tumors originate from the surface epithelium of the ovary. Five major subtypes are included within the surface epithelial group. They are designated as follows: serous, mucinous, endometrioid, clear cell, and transitional cell (or Brenner type). Malignant serous tumors make up one-third of all ovarian serous tumors and approximately half of all malignant ovarian neoplasms [2]. Germ cell tumors are ovarian tumors formed by cells derived from primordial germ cells. Those that differentiate in an extraembryonic (placental or trophoblastic) direction result in yolk sac tumors or choriocarcinomas. Mixed subtypes of germ cell tumors also occur frequently [2]. Choriocarcinomas are germ cell tumors formed by placental elements (namely, trophoblastic cellular elements). Choriocarcinomas are highly malignant and locally invasive tumors that spread extensively throughout the abdominal cavity, and metastasize early. Choriocarcinomas are rare and often are admixed with other germ cell tumors. Primary ovarian choriocarcinomas occur in children and young adults. Choriocarcinomas in the ovary occur as gestational and non-gestational types. Non-gestational choriocarcinomas spread mainly via lymphatics and gestational choriocarcinomas spread primarily via the bloodstream [2]. Non-gestational choriocarcinomas have no paternal chromosomes [12]. It is important to distinguish between these types, as the prognosis and chemotherapeutic response may differ [3, 13]. Sustained remission is achieved in most patients, but it is less common in those with non-gestational choriocarcinomas. Gestational choriocarcinomas may follow decades after an antecedent pregnancy. Our case is unlikely to be of such origin.

The combination of choriocarcinoma and other histological types has been reported in association with some human cancers, predominantly with the female genital tract [14-16] and rarely with carcinomas of the esophagus [17], bladder or renal pelvis [18, 19], stomach [20], rectum [21], lung [22], and breast [23]. The absence of any other malignant germ cell component and the presence of an adjacent serous carcinoma indicate that this was a non-gestational choriocarcinoma arising within a serous carcinoma in this case. Choriocarcinoma arising within an ovarian carcinoma is very rare and only few reports have been documented [3-7]. Recognition of choriocarcinomatous components in ovarian carcinomas is important because of its aggressive behavior [4].

The explanation of association between ovarian carcinoma and choriocarcinoma has been forth as two hypotheses. One hypothesis is that of a true collision tumor between a carcinoma and a choriocarcinoma. This seems to be unlikely in our case. The second hypothesis is that of a divergent differentiation of a serous carcinoma called as “neometaplastic” process or as “retrodifferentiation” or “dedifferentiation” [3, 4, 7]. Such an origin is favored because a transition zone can be identified in some cases as in our case [3, 6].

A diagnosis of choriocarcinoma arising within a serous carcinoma of the ovary needs to be distinguished from the more common occurrence of carcinoma with syncytiotrophoblastic giant cells and osteoclastic giant cells. Ovarian surface epithelial carcinomas can contain scattered trophoblastic cells secreting beta-HCG similar to adenocarcinomas of several other organs (breast, lung, stomach, prostate, and bowel) [4, 7]. In contrast, choriocarcinomas exhibit the typical biphasic cell populations of cytotrophoblasts and adjacent syncytiotrophoblasts and admixed areas of hemorrhage and necrosis as in this case.

The diagnosis of choriocarcinoma arising in an ovarian surface epithelial carcinoma has valid management implications. Chemotherapeutic regimens used in mixed choriocarcinoma and carcinoma are different. There are no established chemotherapeutic regimens [3, 12, 13]. It is logical to use chemotherapeutic agents that target both components [14]. The chemotherapeutic agents used for gestational choriocarcinoma have shown response in non-gestational choriocarcinoma. Alternative chemotherapeutic regimens are used in refractory cases.

Only six cases have been described in the English literature so far. Oliva et al reported two cases of poorly differentiated ovarian carcinoma with choriocarcinomatous components. Immunohistochemical stain for beta-HCG was positive. Extra-abdominal metastases developed early in both patients and, despite multiagent chemotherapy, they died shortly postoperatively [4]. IHC for beta-HCG was negative in our case probably because of post-chemotherapy effects. A case of choriocarcinoma within an ovarian carcinoma was reported by Hafezi-Bakhtiari treated by specific chemotherapy regimen with cisplatin, etoposide, and bleomycin, suitable for both types of malignancy [5]. Our patient was started on metronomic chemotherapy with oral endoxan and topotecan subsequently. Jimenez-Heffernan et al reported a case of ovarian neoplasm in which mucinous cystadenocarcinoma and choriocarcinoma coexisted and blood levels of beta-hCG were elevated. Blood levels of beta-HCG were normal in our case probably because of post-chemotherapy and post-surgery effects [6]. An unusual case of non-gestational choriocarcinoma arising from a primary epithelial ovarian tumor in a 60-year-old woman was described by Oladipo et al. The diagnosis was made retrospectively by reviewing the histology once the patient developed lung metastasis 4 weeks following commencement of single agent carboplatin. Patient was started on POMB/ACE chemotherapy. Though her lung metastasis improved, she subsequently developed brain metastasis and died shortly afterwards [3]. Though there were mediastinal nodes upfront, our patient is free of lung parenchymal or brain metastasis so far. Hirabayashi et al reported a case of admixture of choriocarcinoma of the ovary with three epithelial malignancies treated by primary surgery. The patient died 10 months after the first operation [7]. Our patient had choriocarcinoma admixed with serous carcinoma of the ovary in the extraovarian peritoneal nodules. This has not been described in English literature so far. Mixed carcinoma and choriocarcinoma tumors are biologically aggressive regardless of treatment, and case reports with at least 1 year of follow-up [6] have shown death within 2 years. Presumptive hematogenous spread to the lung is characteristic of choriocarcinoma [3, 4]. In spite of the aggressive histology, the patient is surviving for the past 1 year.

### Conclusion

Non-gestational choriocarcinoma may arise from ovarian carcinoma. The accurate histopathologic diagnosis of these rare mixed malignancies is important, because the prognosis for these malignancies is poor and chemotherapy specific to each histologic component may be chosen. Choriocarcinoma arising within an ovarian carcinoma is very rare and only few reports have been documented. The case described here is an extremely rare presentation, with pure serous carcinoma in the ovary and presence of combination of poorly differentiated serous carcinoma and choriocarcinoma in the peritoneal nodules and omentum.
